# The Emerging Role of Altered Cerebellar Synaptic Processing in Alzheimer’s Disease

**DOI:** 10.3389/fnagi.2018.00396

**Published:** 2018-11-27

**Authors:** Eriola Hoxha, Pellegrino Lippiello, Fabio Zurlo, Ilaria Balbo, Rita Santamaria, Filippo Tempia, Maria Concetta Miniaci

**Affiliations:** ^1^Neuroscience Institute Cavalieri Ottolenghi (NICO), Turin, Italy; ^2^Department of Neuroscience, University of Torino, Turin, Italy; ^3^Department of Pharmacy, School of Medicine, University of Naples Federico II, Naples, Italy; ^4^National Institute of Neuroscience (INN), Turin, Italy

**Keywords:** cerebellum, Alzheimer’s disease, β-amyloid, purkinje cell, synaptic plasticity, noradrenaline

## Abstract

The role of the cerebellum in Alzheimer’s disease (AD) has been neglected for a long time. Recent studies carried out using transgenic mouse models have demonstrated that amyloid-β (Aβ) is deposited in the cerebellum and affects synaptic transmission and plasticity, sometimes before plaque formation. A wide variability of motor phenotype has been observed in the different murine models of AD, without a consistent correlation with the extent of cerebellar histopathological changes or with cognitive deficits. The loss of noradrenergic drive may contribute to the impairment of cerebellar synaptic function and motor learning observed in these mice. Furthermore, cerebellar neurons, particularly granule cells, have been used as *in vitro* model of Aβ-induced neuronal damage. An unexpected conclusion is that the cerebellum, for a long time thought to be somehow protected from AD pathology, is actually considered as a region vulnerable to Aβ toxic damage, even at the early stage of the disease, with consequences on motor performance.

## Introduction

Accumulating evidence indicates that Alzheimer’s disease (AD) is caused by the toxicity of amyloid-β (Aβ) peptide (Haass and Selkoe, 2007; Shankar et al., 2008; Gouras et al., 2010). Aβ peptides are generated from β-amyloid precursor protein (APP) via proteolytic cleavage by the β- and γ-secretases; depending on the site of cleavage, different sized Aβ peptides are generated, with Aβ_1–40_ and Aβ_1–42_ being the most toxic Aβ isoforms (Selkoe, 1998; O’Brien and Wong, 2011). The soluble monomers can assemble into microaggregates, also termed “soluble Aβ oligomers,” protofibrils and fibrils that accumulate in the brains of AD subjects, forming insoluble amyloid plaques. It is now believed that soluble Aβ oligomers are the critical pathogenic molecules in AD leading to synaptic and cognitive dysfunctions (Selkoe, 2008; Ferreira et al., 2015). Accumulation of Aβ peptides and insoluble plaque formation trigger a number of detrimental processes, including hyperphosphorylation of the microtubule-associated protein tau, which leads to the formation of neurofibrillary tangles and neuronal death (Kopeikina et al., 2012; Lasagna-Reeves et al., 2012). In accordance with this hypothesis, recent evidence shows that, in transgenic mice expressing the human tau protein, injection of Aβ_1–42_ monomers can induce tau hyperphosphorylation (Manassero et al., 2016). However, the relevance of this mechanism in the cerebellum is not clear yet. Indeed, the cerebellum exhibits a lower expression of tau compared to the cerebral cortex or to the hippocampus (Hu et al., 2017).

Most of the research in the AD field has been focused on characterizing the toxicity of Aβ on the medial temporal lobe structures, especially the hippocampus, since their dysfunction is considered the main cause of memory loss in AD, the earliest cognitive deficit in this type of dementia (Hyman et al., 1984; West et al., 1994; Scheff et al., 2007; Miniaci and De Leonibus, 2018). However, patients with AD can also develop language, visual or motor symptoms, suggesting that Aβ pathology extends beyond the hippocampus (Caine and Hodges, 2001; Lambon Ralph et al., 2003; Albers et al., 2015).

Studies in both familial (FAD) and sporadic cases of AD have demonstrated that the cerebellum is a vulnerable region in AD patients (Schmahmann, 2016; Jacobs et al., 2018). Elevated levels of Aβ oligomers have been observed in the cerebellar cortex of AD patients (Larner, 1997; Sepulveda-Falla et al., 2011). Furthermore, cases of early-onset FAD, caused by mutation of presenilin-1 (PSEN1; the catalytic subunits of γ-secretase), present Purkinje cell (PC) loss as well as cerebellar deposition of Aβ and high levels of hyperphosphorylated tau (Sepulveda-Falla et al., 2011, 2014). Cerebellar atrophy is another characteristic feature of sporadic AD which affects initially the portions of the cerebellar regions connected to the default mode network, a set of highly interacting brain regions (including angular gyrus, middle temporal gyrus, precuneus and dorsal medial prefrontal cortex) involved in cognition and extensively affected by neurodegeneration such as in AD (Thomann et al., 2008; Mevel et al., 2011; Guo et al., 2016). As the atrophy extends to the cerebellar anterior lobe, patients may display motor dysfunctions, i.e., impairments in gait and limb coordination (Aggarwal et al., 2006).

In the last decades, mouse models of AD expressing mutant human APP, sometimes together with mutant presenilins, have been widely used for investigating the AD pathophysiology and to establish new therapeutic strategies (Ashe and Zahs, 2010; Puzzo et al., 2015; Drummond and Wisniewski, 2017; Sasaguri et al., 2017). Most of the studies on these mouse models have investigated the relationship between Aβ, memory impairment and hippocampal synaptic plasticity, which is considered the cellular correlate of learning and memory (Selkoe, 2002; Haass and Selkoe, 2007; Shankar and Walsh, 2009). Less is known about the effects of Aβ on cerebellar synaptic functions and motor learning. Here, we provide for the first time a comprehensive review of the literature regarding the cerebellar cellular and molecular characterization in mouse models of AD, that can help researchers to better understand cerebellar contribution in neurodegenerative disorders and eventually provide the basis for future research on this field.

## Cerebellar Motor Dysfunctions in Mouse Model of AD

Motor deficits, initially classified as late-onset symptoms in AD patients, have been considered for a long time as irrelevant given the severe deterioration of cognitive functions (Ala and Frey, 1995). However, there is increasing evidence that motor performance is impaired even at the preclinical stage of the disease (Pettersson et al., 2002; Buchman and Bennett, 2011) and a link between motor skills deficits and AD onset probability has also been proposed (Aggarwal et al., 2006).

Significant motor impairment has been also observed in transgenic mice modeling AD (Table 1). For example, APP23 transgenic mice overexpressing APP751 isoform with the Swedish mutation (KM670/671NL) show motor coordination deficit on the rotarod by 3 months of age, before Aβ accumulation (Van Dam et al., 2003). Rotarod motor coordination deficits preceding Aβ accumulation has been also revealed in APP/PSEN1 mice, carrying APP695 isoform with Swedish mutation and mutant presenilin 1 genes (APPswe/PSEN1dE9; Kuwabara et al., 2014). Moreover, APPswe/PSEN1dE9 mice show motor learning deficits on the ErasmusLadder Task (Sepulveda-Falla et al., 2014). Interestingly, in addition to motor coordination deficits on the beam test, TgCRND8 mice, containing human APP695 with the Swedish and Indiana (V717F) mutations, exhibit gait impairment on the footprinting test (Russo et al., 2018).

**Table 1:** Motor behavior and cerebellar electrophysiological phenotypes in murine models of Alzheimer’s disease (AD).

Mouse model	Motor deficits	Cerebellar electrophysiological changes	References
**APPswe/PSEN1-L166P** (Radde et al., 2006)	At pre-plaque stage: No motor coordination and motor learning deficits on balance beam test and accelerated rotarod test No gait impairment on footprinting test	At pre-plaque stage: ↓ PC excitability unaffected PF-PC basal transmission unaffected CF-PC basal transmission ↓ CF-PC paired-pulse depression loss of large mIPSCs	Hoxha et al. (2012)
**APPswe/PSEN1dE9** (Jankowsky et al., 2004)	At pre-plaque stage: Motor coordination deficits on rotarod test Motor learning deficits on the ErasmusLadder task	At pre-plaque stage: unaffected PF-PC basal transmission ↓ LTD at PF-PC synapse	Kuwabara et al. (2014)
		At post-plaque stage: ↓ simple spike firing unaffected complex spike firing	Sepulveda-Falla et al. (2014)
**TgCRND8** (Chishti et al., 2001)	At pre-plaque stage: Motor coordination deficits on balance beam test Gait impairment on footprinting test No motor learning deficits on the accelerated rotarod test	At pre-plaque stage: unaffected PF-PC basal transmission ↓ LTD at PF-PC synapse impaired noradrenergic modulation of PF-PC synapse	Russo et al. (2018)
**APP23** (Sturchler-Pierrat et al., 1997)	At pre-plaque stage: Motor coordination deficits on rotarod test	n.d.	Van Dam et al. (2003)
**5×FAD** (Oakley et al., 2006)	At post-plaque stage: Motor coordination deficits on balance beam test Motor learning deficits on the accelerated rotarod test Gait impairment on footprinting test	n.d.	Ewers et al. (2006) and O’Leary et al. (2018)
**Tg2576** (Hsiao et al., 1996)	At pre-plaque stage: No motor learning deficits on the accelerated rotarod test	n.d.	Dineley et al. (2002)

*Abbreviations: n.d., not determined; PC, Purkinje cell; PF, parallel fiber; CF, climbing fiber; LTD, long term depression*.

On the other hand, several studies report motor deficits only at later stages, when Aβ deposition is already present. For example, 5xFAD mice, carrying human APP695 with Swedish, Florida (APP I716V) and London (V717I) mutations plus PSEN1 with M146L and L286V mutations, exhibit a severe motor impairment at 11–13 months of age consisting of motor coordination and motor learning deficits on the balance beam test and accelerating rotarod, respectively (Ewers et al., 2006). Moreover, 5xFAD transgenic mice show also changes in gait parameters with a shorter stride length respect to wild type (WT) mice on the footprinting test (O’Leary et al., 2018).

Nevertheless, there are other transgenic (Tg) lines that not show motor dysfunctions even in the presence of cerebellar Aβ accumulation, PC death and/or cerebellar electrophysiological changes. These models include Tg2576 mice, which overexpress a mutant form of APP695 with the Swedish mutation (Dineley et al., 2002), and APP/PS1 mice overexpressing APP695 Swedish mutant form plus PSEN1 with the L166P mutation (APPswe/PSEN1-L166P; Hoxha et al., 2012).

These results, taken together, suggest that there is no clear relationship between cerebellar Aβ deposits and motor deficits. It is possible to hypothesize that soluble forms of Aβ, like monomers or oligomers, are more directly related to motor symptoms. This might explain the wide variability of the motor phenotype observed in the different murine models of AD.

Motor deficits are also not correlated to the onset of cognitive impairment. In fact, in TgCRND8 and APPswe/PSdeltaE9 mice, motor deficits precede cognitive symptoms (Chishti et al., 2001; Reiserer et al., 2007). In 5xFAD mice, the cognitive impairment becomes apparent before motor deficits (Oakley et al., 2006). In APP23 mice motor and cognitive symptoms start at the same age (Van Dam et al., 2003).

Given these behavioral outcomes, it is possible that the brain regions engaged in motor and cognitive performances are differentially affected in AD animal models.

## Cerebellar Histopathological Changes

Several studies in AD patients have reported diffuse amyloid plaques in the cerebellum particularly in those with the pre-senile onset of dementia (Larner, 1997). Aβ deposits were found in the molecular layer of the cerebellar cortex extending to the PC layer, and to a lower extent in the granule cell layer (Braak et al., 1989; Sepulveda-Falla et al., 2011). The cerebellar histopathological alterations appear earlier and are more severe in FAD compared to the sporadic form of AD and are characterized by PC loss, neuritic plaques and reactive astrocytosis (Fukutani et al., 1996).

Similar anatomical and temporal patterns of alterations develop in the cerebellum of transgenic mice models of FAD. For example, APPswe/PSEN1dE9 mice show few diffuse Aβ deposits in the molecular layer at 6 months of age; the number of cerebellar deposits increases with disease progression and at higher rate in females vs. males (Lomoio et al., 2012; Kuwabara et al., 2014). At 18/20 months of age, the Aβ plaques appear surrounded by dystrophic neurites, microglia and astrocytes; focal loss of PCs and degeneration of parallel fibers (PFs) have been also observed. Significant loss of PCs (about 60%) has been also revealed in Tg2576 mice (Kozuki et al., 2011); PC loss progresses with animal aging and becomes much more prominent in older transgenic mice. Deep cerebellar nuclei (DCN) neurons appear vulnerable, even at the early stage of disease, in 3xTg-AD mouse model that contains three mutant genes, APPSwe, PS1M146V and tauP301L, leading to both plaque and tangle pathology (Esquerda-Canals et al., 2013). In these mice, loss of DCN is more pronounced at the level of the fastigial nucleus than the interpositus and dentate nuclei.

Taken together these studies suggest that the involvement of the cerebellum is quite variable depending on the mutation, on the region and cell type analyzed. More experiments are needed to understand the role of specific mutations and the involvement of tau in the pathology of animal models of AD. A deeper level of understanding might be obtained by studies of the alterations in cellular function by electrophysiologicaltechniques.

## Alteration of PC Activity and Synaptic Plasticity in Mouse Models of AD

In the cerebellar cortex, elevated levels of soluble Aβ_1–42_ and Aβ oligomers are associated with the reduction of PC intrinsic excitability. Patch-clamp recordings from PCs of 8-month-old APPswe/PSEN1-L166P mice revealed a larger afterhyperpolarization following the first action potential and longer second and third interspike intervals compared to control PCs, which are indicative of higher frequency adaptation (Table 1; Hoxha et al., 2012). No changes of PC membrane excitability were found in 2-month-old APPswe/PSEN1-L166P mice in which the Aβ levels are scarcely detectable. Since PCs are the sole output of the cerebellar cortex, changes in their firing properties can alter the activity of target neurons in the deep cerebellar and vestibular nuclei (Hoxha et al., 2018).

According to different studies, Aβ_1–42_ does not affect the PF-PC basal synaptic transmission (paired-pulse facilitation and/or input-output relationship) whether it is autonomously produced in AD mouse models, including APP/PS1, or administered to cerebellar slices of WT mice, at a concentration of 500 nM (Hoxha et al., 2012; Kuwabara et al., 2014; Russo et al., 2018). Conversely, application of 1 μM Aβ_1–42_ peptides determines a significant decrease in PF-PC synapse activity in cerebellar slices of C57/Bl6 mice (Arbez et al., 2007). Such effect is associated with the decrease of miniature excitatory postsynaptic currents (mEPSCs) amplitude and frequency suggesting an acute effect of Aβ on both presynaptic and postsynaptic compartments. According to the authors, the PF-PC synaptic alterations are caused by the Aβ-induced activation of pro-apoptotic MAP kinases JNK and p38 since their inhibition reversed the negative effect of Aβ on mEPSC amplitude and frequency.

Single-unit extracellular recordings of PCs *in vivo* in the cerebellar lobules (I–V) of 22-week old APPswe/PSEN1dE9 mice revealed a firing frequency reduction of simple spikes, generated by intrinsic membrane properties and PF inputs to PCs, whereas the firing frequency of complex spikes, driven by climbing fiber (CF) inputs, was not affected (Sepulveda-Falla et al., 2014). The CF-PC basal synaptic transmission is also unaffected in cerebellar slices of 8-month-old APPswe/PSEN1-L166P mice, but the paired-pulse depression at the CF-PC synapse is reduced suggesting an altered release of glutamate from CFs (Hoxha et al., 2012).

Interestingly, the PCs of APPswe/PSEN1-L166P mice also showed a selective loss of large amplitude miniature inhibitory postsynaptic currents mediated by gamma-aminobutyric acid (GABA) receptors pointing to a reduction of the GABAergic inhibitory drive on PCs (Hoxha et al., 2012). The decrease of GABA inputs might be considered a compensatory mechanism for the impairment of intrinsic excitability aimed at re-establishing the physiological rate of PC firing. Alternatively, the reduction in membrane excitability might be a homeostatic compensation for the impairment of GABAergic transmission. Dysfunction in the GABAergic signaling system has been also documented in different brain regions of AD patients, including the cerebellum (Mohanakrishnan et al., 1997; Seidl et al., 2001). In particular, a significant loss of GABA content has been found in post-mortem cerebellum samples from AD patients associated with a significant decrease of glutamic acid decarboxylase (GAD), the synthesizing enzyme of GABA (Burbaeva et al., 2014). The results from animal models, together with the immunohistochemical studies on human AD brains, indicate that a GABAergic dysfunction is present in the AD cerebellum, with possible consequences on the complex excitatory/inhibitory balance, thus contributing to the disease pathophysiology (McCormick, 1989).

Although basal synaptic transmission is intact at the PF-PC synapse, cerebellar cortex synaptic plasticity deficits have been observed in AD mice models in correlation with motor learning impairment (Kuwabara et al., 2014). In most cases, the PF- long-term depression (LTD) is impaired by either exogenous synthetic Aβ_1–42_ application or by the abnormally high levels of endogenous Aβ present in AD Tg mouse models. Russo et al. (2018) have recently demonstrated that cerebellar synaptic plasticity is affected early in the time course of the Alzheimer’s pathology. Indeed, electrophysiological recordings on cerebellar slices from 2-month-old TgCRND8 mice revealed a significant alteration of LTD at the PF-PC synapse at the pre-plaque stage. Indeed, TgCRND8 mice exhibit no Aβ plaques before 3-months, but in several brain areas including the cerebellum small amounts of Aβ peptides, which are likely to interfere with synaptic plasticity and learning, have been detected (Chishti et al., 2001; Yu et al., 2014).

These data suggest that, in the cerebellum, deficits of neuronal signaling are present in early stages of AD, and they are paralleled by the onset of motor symptoms. However, the results are sparse and have been obtained in different animal models of AD, so that currently it is difficult to draw conclusions. Future studies are necessary to throw light on the cerebellar neuronal dysfunctions that are involved in motor symptoms of patients with AD. In addition, the activity of cerebellar neuronal networks is under continuous control by diffuse-projection neuromodulatory systems, some of which are severely affected in AD. In the cerebellum, the noradrenergic system is one of the most important neuromodulatory systems.

## Alteration of Noradrenergic Neurotransmission in the Cerebellar Cortex

Noradrenaline (NA) is a well-known neuromodulator involved in a broad variety of brain processes, including attention, arousal, decision making and memory. The cerebellar cortex receives a widespread noradrenergic projection, from the locus coeruleus (LC), which is consistent with the demonstration that the NA is involved in the modulation of cerebellar function including motor learning (Pompeiano, 1998; Cartford et al., 2004; Schweighofer et al., 2004).

Degeneration of LC and loss of noradrenergic innervation in various brain regions have been revealed in AD in correlation with decreased cognitive functions (Bondareff et al., 1987; Bickford, 1993; Grudzien et al., 2007). Studies of post-mortem tissue have demonstrated a significant reduction of total LC cell number in MCI and AD as well as decrease in norepinephrine transporter (NET), responsible for the reuptake of NA, in several brain areas of AD patients including the cerebellum (Tejani-Butt et al., 1993; Gulyás et al., 2010; Kelly et al., 2017). Interestingly, AD patients with aggressive behavior show higher levels of α_2_- and β-adrenergic receptors (ARs) in the cerebellar cortex compared to non-agitated AD subjects, whereas no significant changes in ARs levels were found in prefrontal cortex and hypothalamus (Russo-Neustadt and Cotman, 1997). According to the authors, alterations of cerebellar ARs expression may affect the PC activity and as a consequence, the cerebellar output to the cerebral cortical areas encoding affective and defensive/aggressive behavior.

**Figure 1:**
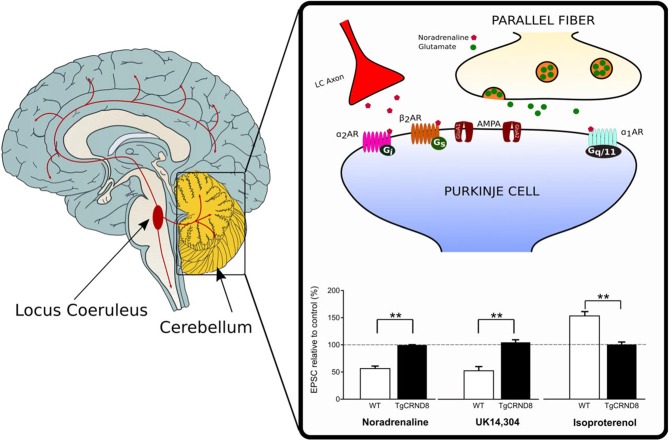
Loss of noradrenergic modulation of the parallel fiber-Purkinje cell (PF-PC) synapse in TgCRND8 mice. In wild-type (WT) mice, the PF-PC excitatory synaptic transmission is depressed by the endogenous agonist noradrenaline (NA) as well as by the α_2_-adrenergic receptor (AR) agonist UK 14,304, but it is potentiated by the β-AR agonist isoproterenol. The same agonists do not induce any effect on PF-excitatory postsynaptic currents (EPSCs) in the cerebellar slices of 2-month-old TgCRND8. Bar graph shows the mean (±SEM) percentage of change of EPSC recorded in PCs of WT and TgCRND8 (Tg) mice. **p < 0.01 Tg vs. WT (modified with permission from Russo et al., 2018).

Depletion of NA has also been reported in the cerebellum of TgCRND8 mice starting at 4–5 weeks of age (Francis et al., 2012). The loss of noradrenergic innervation is likely to contribute to the impairment of cerebellar synaptic function and eventually, motor learning observed in these mice (Russo et al., 2018). Under normal conditions, activation of α-ARs by NA or the α_2_-AR agonist UK 14,304, produces synaptic depression at the PF-PC synapse whereas β_2_-AR activation by isoproterenol facilitates the PF-PC synaptic transmission (Lippiello et al., 2015; Hoxha et al., 2016). On the contrary, application of α- or β-ARs agonist does not induce any effect on PF-EPSCs in the cerebellar slices of 2-month-old TgCRND8 (Figure 1). Furthermore, unlike controls, 2-month-old TgCRND8 mice exhibit a significant impairment of isoproterenol-induced plasticity at the PF-PC in correlation with significant motor coordination and balance deficits (Russo et al., 2018).

Further studies in APP23 transgenic mice have shown that degeneration of LC results in elevated Aβ deposition and increased neuroinflammation in brain areas usually innervated by LC (Heneka et al., 2010). This result is in accordance with the demonstration that NA positively modulates microglia phagocytosis and migration, therefore facilitating Aβ clearance and reducing the inflammatory response to Aβ.

## Mechanisms Involved in Aβ-Induced Neurotoxicity

A general state of inflammation of the central nervous system has been widely documented in AD (Hensley, 2010; Maione et al., 2018). Amongst the elements contributing to the development of neuroinflammation in AD, there are certain cell types, such as astroglia and microglia, that manifest high reactivity and morphological alterations (Akiyama et al., 2000). At the cerebellar level, a severe astrocytosis has been found in the granule layer and white matter of FAD patients, in absence of amyloid plaques (Fukutani et al., 1996). Astrocytosis has been also observed in sporadic AD patients, although the number of astrocytes was significantly lower than in FAD patients.

Further studies in rats have demonstrated that cerebellar astrocytosis can be directly induced by intracerebroventricular administration of Aβ peptides. In particular, Aβ injection determines a significant increase in astrocyte number in the granular and molecular layers (Lee et al., 2014). In addition, Aβ can also activate phagocytosis by microglia in rat mixed granular/glial cell cultures causing the loss of cerebellar neurons (Neniskyte et al., 2011). In the presence of the inhibitor of microglial phagocytosis, L-leucine-methyl-ester, the administration of Aβ_1–42_ (250 nM) does not induce any granule cell loss. However, L-leucine-methyl-ester is not able to prevent granule cell loss at higher concentrations of Aβ_1–42_ (10 μM). This suggests that the loss of neurons induced by low concentrations of Aβ is directly linked to microglial activity, whereas in the presence of higher concentrations of Aβ_1–42_ the cellular loss occurs through a microglia-independent mechanism. According to Neniskyte et al. (2011), the administration of Aβ_1–42_ induces the exposure of phosphatidylserine (PS) on the neuronal surface and activates phagocytosis by microglia. Indeed, in addition to the treatment with phagocytosis inhibitors, antibodies to PS prevented Aβ_1–42_-induced neuronal loss.

Interestingly, an increased secretion of β-amyloid has been assessed in cerebellar granule cells undergoing apoptosis. Aβ can activate signaling cascades responsible for the neurodegeneration of nearby cells; the granular cellular death rate is reduced by anti-Aβ antibodies (Galli et al., 1998).

The apoptotic effect of Aβ_25–35_ on cerebellar granule cells involves the activation of different classes of caspase, including caspase-2, -3 and -6 (Allen et al., 1999). These caspases can cleave tau proteins to generate fragments with toxic activity, such as NH_2_-26–44 tau fragment, which may impair the oxidative phosphorylation leading to the accumulation of reactive oxygen species (ROS; Atlante et al., 2008). The excessive presence of free radicals and ROS in the nucleus determines the oxidation of nucleic acids and, consequently, harmful mutations and cellular death. An increase in 8-OH-adenine, 8-OH-guanosine and fapyadenine in nuclear DNA has been reported in the cerebellar cortex of patients in the last stage of AD (Wang et al., 2005). The oxidative stress is associated with an increase of redox-active iron at the early preclinical stage of AD. The redox metal accumulation affects PCs less than other cerebellar cell types, including glial cells, likely due to the different redox environment within the cerebellum (Smith et al., 2010).

In TgCRND8 mice, accumulation of Aβ is accompanied by an increase of oxidative stress (Yu et al., 2014). The same mice show an increased cerebellar NADPH oxidase activation at pre-plaque stage accompanied by enhanced phospholipid peroxidation compared to WT mice (Russo et al., 2018). It should be pointed out that the oxidative stress level in the cerebellum is lower than in the cortex and this may be correlated with the differences in the antioxidant activity and levels of trace metals including iron.

## Conclusions

In this review article, by exploiting the knowledge derived from single and original studies, the involvement and contribution of the cerebellum in AD are outlined for the first time. According to these studies, the cerebellar involvement by AD pathology is more pronounced than previously thought. Aβ cerebellar deposits increase with disease progression in the cerebellar cortex of both patients and mouse models of AD, leading to plaque formation and loss of PCs. Further investigation on Tg mice has recently provided important insight into the effects of Aβ on cerebellar synaptic transmission and plasticity at the early stage of disease, that may lead to motor coordination and learning deficit. Such synaptic and motor learning alterations are in part mediated by deficits in NA signaling. The *in vitro* approach has also uncovered the role of cerebellar astrocytes and microglia phagocytosis as well as oxidative stress in the Aβ-induced neuronal damage.

Given the role of the cerebellum in motor and cognitive processes, additional studies must be undertaken to understand how neurodegenerative changes that develop progressively in this brain structure impact the progression of AD from the earliest to the final stages of the disease.

## Author Contributions

MM and FT designed the outline of the article. EH, PL, FZ, IB, RS, FT and MM wrote the manuscript. IB and PL prepared the figure.

## Conflict of Interest Statement

The authors declare that the research was conducted in the absence of any commercial or financial relationships that could be construed as a potential conflict of interest. The reviewer ET declared a shared affiliation, with no collaboration, with several of the authors, EH, IB, FT, to the handling editor at time of review.
